# Placental Endoplasmic Reticulum Stress and Oxidative Stress in the Pathophysiology of Unexplained Intrauterine Growth Restriction and Early Onset Preeclampsia

**DOI:** 10.1016/j.placenta.2008.11.003

**Published:** 2009-03

**Authors:** G.J. Burton, H.-W. Yung, T. Cindrova-Davies, D.S. Charnock-Jones

**Affiliations:** a Centre for Trophoblast Research, Department of Physiology, Development and Neuroscience, University of Cambridge, UK; b Department of Obstetrics and Gynaecology, University of Cambridge, UK

**Keywords:** Endoplasmic reticulum stress, Oxidative stress, Intrauterine growth restriction, Preeclampsia, Placental pathology

## Abstract

The pregnancy complications of unexplained intrauterine growth restriction and early onset preeclampsia are thought to share a common aetiology in placental malperfusion secondary to deficient maternal spiral artery conversion. A key question is whether the contrasting clinical manifestations reflect different placental pathologies, or whether they are due to altered maternal responses to a common factor derived from the placenta. Recently, molecular evidence of protein synthesis inhibition secondary to endoplasmic reticulum stress has provided an explanation for the small placental phenotype in both conditions. However, other pathways activated by more severe endoplasmic reticulum stress are only observed in placentas from pregnancies associated with early onset preeclampsia. Here, we review the literature and conclude that there is evidence of greater maternal vascular compromise of the placenta in these cases. We speculate that in cases of normotensive intrauterine growth restriction the placental pathology is centred predominantly around endoplasmic reticulum stress, whereas in cases complicated by preeclampsia oxidative stress is further superimposed. This causes the release of a potent mix of pro-inflammatory cytokines, anti-angiogenic factors and trophoblastic aponecrotic debris into the maternal circulation that causes the peripheral syndrome. Maternal and fetal constitutional factors may modulate how the placenta responds to the maternal vascular insult, and how the mother is affected by the placental factors released. However, the principal conclusion is that the difference between these two conditions lies in the severity of the initiating deficit in spiral arterial conversion, and the relative degrees of endoplasmic reticulum stress and oxidative stress induced in the placenta as a result.

## 1 Introduction

Unexplained intrauterine growth restriction (IUGR), defined as a failure of the fetus to reach its genetic growth potential, and early onset preeclampsia are often considered together as consequences of ‘placental insufficiency’. Their clinical manifestations are very different, however, and so comparison of the placental changes in the two conditions may shed important light on the elusive pathophysiological mechanisms underlying preeclampsia. A spectrum of possibilities exists. At one extreme one might hypothesise that the placental changes are identical and that the contrasting clinical presentations are due solely to differences in the maternal susceptibility to products emanating from a stressed placenta. Early reports suggested that women who develop early onset preeclampsia display pre-existing, but subclinical, risks for cardiovascular disease, but later studies showed these risks to be shared by women who develop normotensive IUGR (reviewed in Ref. [1]). In a variation of this theme, Ness and Sibai recently postulated that it is the presence of maternal metabolic syndrome that triggers the onset of preeclampsia in conjunction with abnormal placentation [1]. At the other extreme one might hypothesise that the placental changes are different in the two conditions, and that these determine the contrasting clinical outcomes.

Here, we review recent data supporting the latter hypothesis, although we recognise that the two scenarios are not mutually incompatible and that it is likely that both maternal and placental factors may contribute to differing degrees in individual cases. Evidence is drawn from the literature, and from our own recent data. Within the older literature the terms IUGR and ‘small for gestational age’ (SGA) were often used interchangeably, although it is now realised that the two are not synonymous. Not all SGA babies identified from centile distributions are growth restricted, and the use of Doppler velocimetry to assess uterine arterial resistance, and of ultrasound to compare cranial and abdominal growth trajectories, has led to a more precise diagnosis of IUGR. Equally, the distinction between early- and late-onset preeclampsia has not always been made. When reviewing studies we have, wherever possible, drawn on those in which there is clear evidence of true IUGR or early onset preeclampsia, and pointed out those in which doubt exists.

## 2 Spiral arterial conversion

Reduced trophoblast invasion, resulting in deficient conversion of the uterine spiral arteries, has long been implicated in the causation of IUGR and preeclampsia. There is general agreement that it is the myometrial segments of the arteries that are most severely affected, and that there is a gradation between IUGR cases that are normotensive and hypertensive (IUGR + PE) [2–5]. Thus, Khong et al. reported that physiological changes were present in the decidual segments of approximately 60% of placental bed biopsies from cases of SGA (defined as birth weight <10th centile) compared with 20% in the myometrial segments. The equivalent values for SGA + PE samples were 40% and 0% respectively, whereas in normal controls physiological changes were seen in both segments of all arteries [6]. A similar gradation was also found by Gerretsen et al. at the endometrial/myometrial junction. In addition, these authors observed a strong positive correlation within the SGA population alone between the birth weight centile and the presence of physiological changes [3].

In many descriptions of uteroplacental blood flow it is stated that deficient spiral artery conversion is associated with reduced placental perfusion. It is not clear, however, how dilation of the terminal segment of an artery can have a major impact on the volume of flow through the vessel. Whilst it is true that the funnel-like dilatation will reduce the local resistance to blood flow, the length of the dilated segment is only 2–3 mm [7], and so the contribution to the overall uterine arterial resistance is likely to be very small. Indeed, if resistance is a key factor one might ask why the changes associated with physiological conversion do not extend further towards, or even into, the arcuate arteries. The answer may lie in the fact that the segment of the spiral artery just below the endometrial/myometrial junction is unique in that it is particularly contractile and responsive to endocrine stimuli [8,9]. Contraction of this segment is thought to limit blood loss at the time of menstruation, but whilst this may be an advantage during the non-pregnant cycle it poses a danger to feto-placental wellbeing if it occurs during pregnancy. Hence, we have speculated that one of the principal functions of physiological conversion is to remove the smooth muscle from this segment, ensuring uninterrupted flow to the placenta [10]. Failure to do so would place the placenta at increased risk of ischaemia–reperfusion-type insult, which is a powerful stimulus for the generation of oxidative stress [11].

A second mechanism by which deficient spiral artery conversion may predispose the placenta to malperfusion is through its association with acute atherotic changes. These changes are characterised by the presence of lipid-laden mononuclear cells that form intimal plaques [12,13]. The plaques project into the vessel lumen, and so may restrict uteroplacental blood flow depending on their size. Whilst there seems agreement that these lesions are common in the decidual segments of spiral arteries in cases of IUGR + PE, their association with IUGR alone is contentious. In a small study Sheppard and Bonner reported acute atherotic changes in both normotensive and hypertensive cases of SGA [13]. By contrast, Brosens et al. examined a larger collection of samples and did not detect the lesion in any normotensive pregnancy, whether SGA (defined as birth weight <10th centile) was present or not [2]. They did, however, observe the lesion in 9/24 placental bed biopsies from cases of SGA + PE. Reconciling these conflicting claims is difficult, given the limitations imposed by the sampling techniques employed and the fact that *ex vivo* collapse of the vessels gives a very false impression of their calibre *in vivo*.

Nonetheless, the morphological data suggest that the maternal circulation to the placenta is compromised to a greater degree in cases of early onset preeclampsia associated with growth restriction than in IUGR alone. This conclusion is consistent with the finding from a prospective study that mean birth weight is lower in pregnancies complicated by IUGR secondary to preeclampsia (2164 g) than in cases of unexplained IUGR (2555 g) [14]. Similarly, placental villous and capillary surface areas tend to be lower in cases of IUGR + PE than IUGR alone, although the differences were not statistically significant when these groups were compared against normal controls and cases of late-onset preeclampsia, where the values are almost twice as large [15].

One might expect, therefore, that the placental pathology may be more severe in cases of IUGR + PE than in IUGR alone. Vascular compromise can affect the activity of a wide range of cell organelles. In the past, much attention has been paid to the syncytiotrophoblastic mitochondria, and their role in inducing apoptosis [16–18]. Here, we focus on the endoplasmic reticulum, which is the site of synthesis and processing of all secretory and membrane-bound proteins.

## 3 Endoplasmic reticulum stress

Endoplasmic reticulum (ER) stress has recently been identified as a major regulator of cell homeostasis through its involvement in post-translational protein modifications and folding, and its capacity to activate the unfolded protein response (UPR) [19–21]. Normal folding requires that unique conditions be maintained within the ER lumen, and nascent proteins are initially bound to Ca2^+^-dependent chaperone proteins, such as glucose-regulated protein 78 (GRP78 or BiP). To enable these chaperones to function correctly the ER lumen contains a very high concentration of Ca^2+^ ions, maintained by active transport through Ca^2+^ ATPases. The lumen is also an oxidative environment, critical for the formation of disulphide bonds. Disturbance of these conditions leads to accumulation of misfolded proteins within the lumen, triggering the evolutionarily conserved UPR. The UPR aims to restore homeostatic balance within the ER, but if this cannot be achieved it activates the apoptotic machinery. Because of the high energy requirements of the ER, the UPR can be activated by relatively minor metabolic disturbances.

**Fig. 1:**
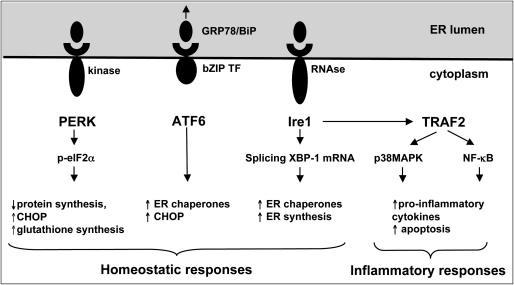
Diagrammatic representation of the signalling pathways activated in the unfolded protein response following ER stress. The sensor molecules, PKR-like endoplasmic reticulum kinase (PERK), inositol-requiring 1 (Ire1) and activating transcription factor 6 (ATF6), are transmembrane proteins normally held inactive by the binding of GRP78/BiP, but are released when the GRP78 preferentially binds to misfolded proteins accumulating in the ER lumen. The UPR aims to restore homeostasis within the ER, but there are also links to the inflammatory response through the Ire1 pathway and TNF receptor-associated factor 2 (TRAF2).

The UPR comprises three principal signalling pathways that have overlapping functions. The sensor molecules, PKR-like endoplasmic reticulum kinase (PERK), inositol-requiring 1 (Ire1) and activating transcription factor 6 (ATF6), are transmembrane proteins whose N-termini project into the ER lumen (Fig. 1). Normally these sensors are held inactive through binding of GRP78 to their N-terminus, but withdrawal of this chaperone by competitive binding to accumulating misfolded proteins causes dimerisation, autophosphorylation and activation of PERK and Ire1. Activation of PERK results in the phosphorylation of eukaryotic initiation factor 2 subunit α (eIF2α), rapidly blocking protein translation and reducing the protein burden within the ER. Ire1 contains an endoribonuclease domain, and when activated splices XBP-1 pre-mRNA to produce a variant encoding the 41 kDa XBP-1 protein, a bZIP-family transcription factor. XBP-1 activates transcription of genes regulating the breakdown of misfolded proteins, and ER biogenesis. When ATF6 is freed from GRP78 it translocates to the Golgi where it is cleaved to form a transcription factor that promotes expression of ER chaperone genes. In severe cases, the PERK and ATF6 pathways lead to increased expression of CHOP, a pro-apoptotic protein [19,20].

**Fig. 2:**
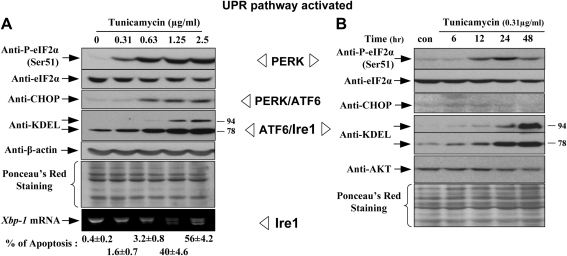
An illustration of the fact that the different signalling pathways comprising the UPR can be activated separately, and at different levels of ER stress, following treatment of JEG-3 cells with tunicamycin. A) Exposure to increasing doses of tunicamycin for 24 h; B) exposure to a low dose (0.31 μg/ml) for 6–48 h. Adapted from Ref. [24] with permission of the American Society of Investigative Pathology.

Because the UPR is a homeostatic mechanism one would expect teleologically to see a graded response in activation of signalling pathways consequent upon ER stress. This is indeed what we observed when JEG-3 choriocarcinoma cells were exposed to incremental doses of tunicamycin, which blocks glycosylation within the ER and is a potent inducer of the UPR. Thus, at low doses there was increased phosphorylation of eIF2α and increased GRP78, at more moderate doses activation of CHOP, and at high doses an increase in GRP94 (Fig. 2A). The rate of apoptosis in the cells correlated closely with the expression of CHOP. Equally, in a time-course experiment using a low sublethal dosage of tunicamycin we observed increased phosphorylation of eIF2α and increased GRP78 and 94, but no activation of CHOP, indicating that the latter is an endstage phenomenon (Fig. 2B). It was also notable that these cells proliferated at a slower rate than untreated controls.

Because ER stress can be induced in other systems by vascular malperfusion [22] we examined placentas from cases of IUGR and IUGR + PE for evidence of activation of the UPR. We restricted the analyses to placentas delivered by caesarean section, as our previous work had shown that stress-response signalling pathways are activated strongly in the placenta following labour [23]. Increased phosphorylation of eIF2α was observed in both sets compared to normal controls, and to the greatest extent in the IUGR + PE placentas. Consequently, the levels of many kinases, including those of the AKT/mTOR pathway, were reduced, leading to multiple blocks to translation initiation [24]. One of the proteins particularly affected by the UPR in other systems is cyclin D1 [25], and levels were severely reduced in our IUGR and IUGR + PE placentas compared to normal controls. A lower rate of cell proliferation could explain the placental phenotype in IUGR, and our *in vivo* and *in vitro* results are consistent with a recent report of a lower frequency of cytotrophoblast cells immunopositive for Ki67 antigen in cases of severe IUGR [26]. The central importance of these pathways to placental growth is illustrated by the fact that in the mouse knockout of *Akt1* alone causes placental and late-onset fetal IUGR [27].

Further evidence that the degree of ER stress was greater in the IUGR + PE placentas than in the IUGR alone cases was provided by the fact that levels of GRP94 and CHOP were significantly raised in former, but not in the latter [24]. CHOP is a transcription factor that inhibits expression of BCL-2, and so is pro-apoptotic. Several studies have estimated the frequency of apoptosis in placentas from complicated pregnancies, but have reported widely differing values depending on the techniques employed. Indices for nuclei of all cell types of 0.17%, 0.24% and 0.39% have been reported for normal, IUGR and IUGR + PE placentas respectively on the basis of light and transmission electron microscopic appearances [28,29]. Similar values were obtained for normal and preeclamptic placentas using the TUNEL technique by Allaire et al. [30]. By contrast, Ishihara et al. obtained values of approximately 1%, 4% and 8% in the syncytiotrophoblast alone on the basis TUNEL labelling [31]. Despite these differences in absolute values there is general agreement that apoptosis is increased in IUGR placentas, and even more so in those from cases of IUGR + PE. This increase in cell death could also contribute to the smaller placental phenotype, but more importantly may underlie the shedding of trophoblastic microparticles that occurs in preeclampsia but not in IUGR alone [32]. This debris has been implicated in the activation of the maternal endothelial cells that characterise the maternal syndrome [33].

To conclude, ER stress and evidence of the UPR are observed in both IUGR and PE + IUGR placentas, but to a greater extent in the latter. The consequent reduction in cell proliferation, coupled with increased rates of apoptosis, provides a plausible and sufficient explanation for the small placental phenotype of IUGR. But can the difference in degree of ER stress explain the enhanced inflammatory response that underpins the maternal syndrome of preeclampsia and distinguishes the clinical manifestations of these two conditions? The increased trophoblastic apoptosis observed in IUGR + PE is a strong potential factor through its association with microparticulate debris. Another possibility is the close relationship between ER stress and oxidative stress, and the additional contribution that the latter may make to the pathophysiology.

## 4 Placental oxidative stress and the inflammatory response

There are three principal ways by which ER and oxidative stress may be linked. Firstly, if sufficiently severe the same vascular stimulus may give rise to both stresses. Thus, ischaemia–reperfusion and hypoxia may precipitate ER stress through perturbations of calcium homeostasis, but can also lead to increased generation of reactive oxygen species (ROS) through mitochondrial pathways or by proteolytic cleavage of xanthine dehydrogenase to the xanthine oxidase form [34]. Activity of the latter is notably higher in preeclamptic placentas than in normal controls [35].

Secondly, protein folding is an oxidative event that itself generates ROS [36]. A high secretory burden or repeated attempts to refold misfolded proteins may therefore lead to increased intracellular concentrations of ROS.

Thirdly, the UPR can activate some of the same intracellular inflammatory signalling pathways as oxidative stress [19,37]. Ire1 contains a Ser/Thr kinase domain in addition to its endoribonuclease domain, and is capable of activating the NF-κB pathway through phosphorylation of IKK, and the p38MAPK pathway through ASK1 (Fig. 1). Furthermore, activation of NF-κB can arise through the inhibition of protein translation secondary to activation of PERK, as the half-life of IKK is shorter than that of NF-κB [37].

We have recently demonstrated that exposing term placental explants to hypoxia-reoxygenation *in vitro* is a powerful generator of oxidative stress [11], and stimulates increased secretion of pro-inflammatory cytokines, such as TNFα and IL-1ß, and anti-angiogenic factors, such as the soluble receptor for vascular endothelial growth factor (sFLT-1) [23,38]. It also results in increased trophoblastic apoptosis, and the release of free fetal DNA [17,39]. These changes can be effectively blocked by addition of the antioxidant vitamins C and E, or inhibitors of the p38 and NF-κB pathways [23,40].

All these factors released from villous explants have been implicated in the pathophysiology of preeclampsia, and levels are different in cases of IUGR + PE than in IUGR alone (Table 1). Is it possible, therefore, that the additional presence of placental oxidative stress accounts for the different clinical presentations of IUGR and IUGR + PE?

## 5 The contribution of placental oxidative stress to preeclampsia

There is extensive evidence of placental oxidative stress in preeclamptic placentas [41,42], and it is generally believed that this plays a key intermediary role in generation of the syndrome [43,44]. In contrast, little attention has been paid to placentas from cases of IUGR alone. In one of the few studies to compare IUGR and IUGR + PE placentas no difference in the levels of 4-hydroxynonenal, a product of lipid peroxidation, was detected immunohistochemically between IUGR and preeclamptic placentas [45]. Equally, however, the levels were no different from those in normal control placentas, but this negative result may reflect the mode of delivery of the placentas studied. The authors did not state whether these were delivered vaginally or by caesarean section, but it is now appreciated that labour induces significant placental oxidative stress and lipid peroxidation [46]. Nonetheless, levels of oxidative DNA damage, as detected by antibodies against 8-hydroxy-2′-deoxyguanosine, were higher in IUGR (*P* = 0.012) and IUGR + PE placentas (*P* = 0.0021) than in normals, with a trend towards being greatest in the latter [45].

In another study examining placental antioxidant defences a reduced level of the mRNA encoding glutaredoxin was found in IUGR + PE placentas, whereas the level in IUGR was the same as in normal controls delivered by caesarean section [47]. The level of thioredoxin mRNA was reduced in both IUGR and IUGR + PE placentas compared to the controls, with a trend to a greater reduction in the latter.

These results are consistent with more recent data showing an increase in the level of the hypoxically regulated protein NDRG1 (N-myc downstream-regulated gene 1) at both the protein and the mRNA levels in IUGR + PE compared to IUGR alone [48]. Increased expression was also observed in normal placentas following labour and vaginal delivery, suggesting that malperfusion is an adequate stimulus for its upregulation. A difference in expression of hypoxically regulated genes between the two groups was also observed by Vaiman et al. using suppression/subtraction hybridisation technologies [49]. While specific gene profiles could be identified for IUGR and IUGR + PE caesarean delivered placentas, only those genes within the latter's grouping were identified as being hypoxically regulated.

Thus, although detailed comparisons of oxidative stress have not been made between non-laboured IUGR and IUGR + PE placentas, the available data do suggest that stress is greater in the latter.

## 6 Overview

**Fig. 3:**
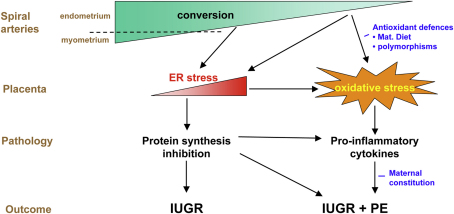
Diagrammatic representation of how placental ER stress and oxidative stress may contribute to the pathophysiologies of IUGR and IUGR + PE. We speculate that in IUGR alone the pathology is predominantly based around ER stress, with homeostatic responses, in particular protein synthesis inhibition, being responsible for the small placental phenotype. When maternal vascular compromise is more severe placental oxidative stress may be superimposed, causing the additional release of a cocktail of pro-inflammatory factors that result in the peripheral syndrome of preeclampsia.

From this review it will be appreciated that there are many common features in the placental changes seen in unexplained IUGR and IUGR + PE, and that the differences are mostly a matter of degree. We speculate that the two conditions represent different points along a spectrum of placental pathologies secondary to deficient spiral artery conversion (Fig. 3). Minor deficiencies in arterial conversion may lead to low grade fluctuations in villous oxygenation that cause homeostatic responses in the form of mild ER stress. It is probable that these occur from the time of onset of the maternal circulation at the start of the second trimester. Protein synthesis inhibition will result in reduced cell proliferation, leading to the small placental phenotype. Whether translation inhibition also causes the reduced levels of System A amino acid transporters that are characteristically seen in IUGR placentas [50] remains to be determined. Potentially, this loss of transporter activity could compound the fetal growth restriction induced by the small placental phenotype, although interestingly System A transporter activity is normal in SGA + PE placentas [51].

More major deficiencies in physiological conversion will cause more severe, and perhaps more frequent, periods of placental ischaemia–reperfusion through greater spontaneous vasocontractility. The distal parts of the spiral arteries will also be exposed to these fluctuations in flow, and this most probably accounts for the development of secondary atherotic lesions in these segments. The lesions will occlude the arterial lumens to a greater or lesser degree depending on their size, causing a more permanent reduction in maternal placental blood flow. We speculate that the greater severity of vascular insult experienced by the placenta in such cases superimposes oxidative stress on top of the pre-existing ER stress. Consequent upon this there is stimulation of stress-response pathways, resulting in the release of pro-inflammatory cytokines into the maternal circulation. Whether this is an adaptive response aimed at increasing maternal blood pressure and so improving placental perfusion remains to be determined. However, under the most severe stress the endpoint of all these signalling pathways is activation of the apoptotic cascade. As a result a potent cocktail of pro-inflammatory cytokines, anti-angiogenic factors and trophoblastic apoptotic debris is released into the maternal circulation, where the components combine to activate the maternal endothelial cells and so cause the peripheral syndrome of preeclampsia (Fig. 3).

This model fits with existing data and provides further testable hypotheses. When considering the model it should be remembered that the definitions of IUGR and IUGR + PE are artificial clinical constructs, and that both birth weight and maternal blood pressure vary as a continuum across patient groups. A spectrum of placental pathology and maternal responses is therefore to be expected. It should also be remembered that there are potentially critical interactions between maternal and fetal constitutional factors and the magnitude of the two stress responses. Hence, maternal diet, in particular her intake of micronutrients, may play a key role in determining the efficacy of the placental antioxidant defences. Fetal polymorphisms that affect activity of the principal antioxidant enzyme defences may also be important. Finally, the maternal response to the cocktail of placental factors may be heavily influenced by the presence of dyslipidaemia or the metabolic syndrome. Despite these caveats, there is increasing evidence that the placental pathophysiology is different in the two conditions, and that this most probably reflects a gradation in the severity of the initiating maternal vascular compromise.

## 7 Conflict of interest

The authors do not have any potential or actual personal, political, or financial interest in the material, information, or techniques described in this paper.

## Figures and Tables

**Table 1:** A summary of changes observed in pregnancies complicated by IUGR and IUGR + PE compared to normal controls.

Variable	IUGR	IUGR + PE	
Spiral artery conversion	↓	↓↓	[3,6]
Spiral artery atherosis	↑	↑↑	[2]
Villous vol. at term	↓↓	↓↓	[15]
Birth weight	↓	↓↓	[13]
Hypoxia-activated genes	Normal	↑	[49]
Placental NDRG1 expression	↑	↑↑	[48]
DNA damage	↑	↑	[45]
Syncytiotrophoblast apoptosis	↑	↑↑	[28–30]
Maternal serum STBM	Normal	↑	[32]
Maternal serum sFLT	Normal	↑	[52]
	↑	↑↑	[53]
Maternal serum PlGF	↓	↓	[52]
Maternal urinary PlGF	Normal	↓↓	[54]
Maternal serum cell-free fetal DNA	Normal	↑	[55]
Maternal serum leptin	Normal	↑	[56]
Maternal serum TNF-α, IL-6, IL-8	Normal	↑↑	[57]
